# Cerebrospinal Fluid in a Small Cohort of Patients with Multiple Sclerosis Was Generally Free of Microbial DNA

**DOI:** 10.3389/fcimb.2016.00198

**Published:** 2017-01-06

**Authors:** Juan Jovel, Sandra O'keefe, Jordan Patterson, Michael Bording-Jorgensen, Weiwei Wang, Andrew L. Mason, Kenneth G. Warren, Gane Ka-Shu Wong

**Affiliations:** ^1^Department of Medicine, University of AlbertaEdmonton, AB, Canada; ^2^Department of Physiology, University of AlbertaEdmonton, AB, Canada; ^3^Department of Biological Sciences, University of AlbertaEdmonton, AB, Canada; ^4^Beijing Genomics Institute-Shenzhen, Beishan Industrial ZoneShenzhen, China

**Keywords:** multiple sclerosis, cerebrospinal fluid, microbiome, metagenomics, bioinforamtics

## Abstract

Multiple sclerosis (MS) is a common cause of non-traumatic neurologic disability with high incidence in many developed countries. Although the etiology of the disease remains elusive, it is thought to entail genetic and environmental causes, and microbial pathogens have also been envisioned as contributors to the phenotype. We conducted a metagenomic survey in cerebrospinal fluid (CSF) from 28 MS patients and 15 patients suffering other type of neurological conditions. We detected bacterial reads in eight out of the 15 non-MS patients and in a single MS patient, at an abundance >1% of total classified reads. Two patients were of special interest: one non-MS patient harbored ~73% bacterial reads, while an MS patient had ~83% bacterial reads. In the former case, *Veillonella parvula*, a bacterium occasionally found associated with meningitis was the predominant species, whilst *Kocuria flava*, apparently an environmental bacterium, predominated in the latter case. Thirty-four out of 43 samples contained <1% bacterial reads, which we regard as cross- or environmental contamination. A few viral reads corresponding to Epstein-Barr virus, cytomegalovirus, and parvovirus were also identified. Our results suggest that CSF of MS patients is often (but not always) free of microbial DNA.

## Introduction

Multiple sclerosis (MS) is a common inflammatory-demyelinating disease of the central nervous system (CNS), which leads to impaired sensory, motor, cerebellar, brain stem, and autonomic functions, and is the foremost cause of non-traumatic neurologic disability in young and middle age patients (Giesser, 2011). At the molecular level, demyelination involves deterioration of myelin sheaths that ensheathe axonal connection between neurons, primarily by macrophages (Comi, 2010). Because T cells mediate myelin degradation, MS is often referred to as an autoimmune disease (Piccio and Cross, 2011). Despite intense research, the cause of MS remains elusive, but the evidence accumulated so far points toward a multifactorial etiology, including genetic (Oksenberg, 2013; Cree, 2014; Sawcer et al., 2014; Axisa and Hafler, 2016) and environmental (O'Gorman et al., 2012; Ascherio, 2013; Malli et al., 2015) factors. In Caucasian cohorts, MHC haplotypes have been found strongly associated with MS (McFarland and Martin, 2007). Some environmental predisposing factors have also been identified, including vitamin D deficiency, smoking, and women are twice as susceptible as men, possibly due to a neuroprotective role of testosterone (Gold and Voskuhl, 2009). Although the disease is distributed worldwide, it holds a positive correlation with latitude, and foci of high incidence are rather patchy across the globe, suggesting that some ethnic groups are more susceptible than others (Ramagopalan and Sadovnick, 2011; Pantazou et al., 2015) or that locally-concentrated infectious microbes may be associated with disease biogenesis (Comi, 2010; Libbey et al., 2014). Furthermore, in a large cohort of patients (*n* > 40,000) from Canada, Great Britain, Denmark, and Sweden, it was found that significantly fewer people suffering MS were born in November, while significantly more MS patients were born in May, which might point to seasonal oscillations in vitamin D or infectious agents, which in turn may interact with genetic factors since the effect was heightened in familial cases (Willer et al., 2005).

Cerebrospinal fluid (CSF) is a clear, non-viscous, material with a very low content of proteins, potassium, and glucose, when compared to plasma, but higher content of chloride and sodium, which maintains CSF neutral charge (Davson and Segal, 1996). It is believed to originate predominantly as a secretion of the choroid plexuses in the ventricles of the brain, upon filtering of plasma through choroid epithelial cells (Davson and Segal, 1996), although this hypothesis has been challenged and instead was proposed that CSF also originates in other compartments of the CSF system (Orešković and Klarica, 2016). CSF localizes to the brain ventricles and subarachnoid space and also surrounds the spinal cord (Davson and Segal, 1996). In addition to mechanical cushion or buoyancy for the cortex, CSF also provides immunological protection to the brain (Nathanson and Chun, 1989), chemical stability (Praetorius, 2007), among other functions.

The presence of immunoglobulins (IgG) in CSF in a large proportion of patients undergoing neurological disorders suggests that at least some of those diseases might have an infectious etiology (reviewed in Das Sarma, 2010). For instance, IgG with specificity against measles virus was found in patients suffering subacute sclerosing panencephalitis (Connolly et al., 1967; Connolly, 1968); similarly, antibodies against Cryptococcus have been found in CSF of meningitis patients (Porter et al., 1977). A wide range of microorganisms has been proposed as putative cause for MS (Johnson, 1994; Swanborg et al., 2003; Stuve et al., 2004; O'Gorman et al., 2012; Zawada, 2012; Libbey et al., 2014). Many studies have also reported the association of Herpes viruses and MS (Warren et al., 1977; Merelli et al., 1997; Moore and Wolfson, 2002; Kuusisto et al., 2008), and the gut microbiome of MS patients exhibit a different profile than that from paired control subjects (Chen et al., 2016; Jangi et al., 2016). It is not clear how antigen-presenting cells in the CNS can interact with activated lymphocytes in the periphery, but it has been proposed that the choroid plexus may display antigens in CSF, likely presented by astrocytes or glial cells, to peripheral blood cells through the choroid epithelium (Nathanson and Chun, 1989).

Since MS is a demyelinating disease, logic would suggest that potential pathogens influencing the disease should also be able to induce demyelination. Several species of bacteria have been reported invading the CNS (Casserly et al., 2007), including *Mycoplasma pneumoniae* (Abramovitz et al., 1987) and *Chlamydia pneumoniae* (Sriram et al., 1999). *M. pneumoniae* reportedly induces demyelination (Greenlee and Rose, 2000) but this finding has not been replicated by other investigators (Casserly et al., 2007; Lindsey and Patel, 2008). Another hypothesis emerging from animal models suggests that bacterial superantigens (e.g., *Staphylococcus aureus* enterotoxins A, B, C, D, and E) activate auto-reactive T cells, which then promotes the onset of immune diseases like MS (Brocke et al., 1993). Indeed, a significant proportion of patients with relapsing MS (within 30 days) were positive for *S. aureus* enterotoxin A as compared to subjects without MS (Mulvey et al., 2011). For viruses, several mechanisms of demyelination have been proposed, including viral lysis of infected oligodendrocytes or immune lysis of uninfected oligodendrocytes specifically or non-specifically triggered by the viral infection (reviewed in Libbey et al., 2014). While microbes are attractive candidates for triggering MS, the environmental triggers for disease have yet to be determined, reflecting limited sensitivity of techniques applied for microbial detection as well as concomitant problems of contamination of samples. Accordingly, we sought to analyze the composition of DNA circulating in CSF from patients affected by MS or other neurological conditions, using an unbiased metagenomics approach to identify possible microbial triggers of MS. Our main findings suggest that CSF is often a microbial DNA free environment, but occasionally can be loaded with bacterial DNA. Virus-like sequences were initially detected, but upon careful examination most sequences turned out to be spurious hits belonging to contaminating DNA. A limited fraction of the reads may correspond to *bona fide* herpesviruses and parvoviruses.

## Results

### Most multiple sclerosis (MS) samples are free of bacterial DNA

**Figure 1:**
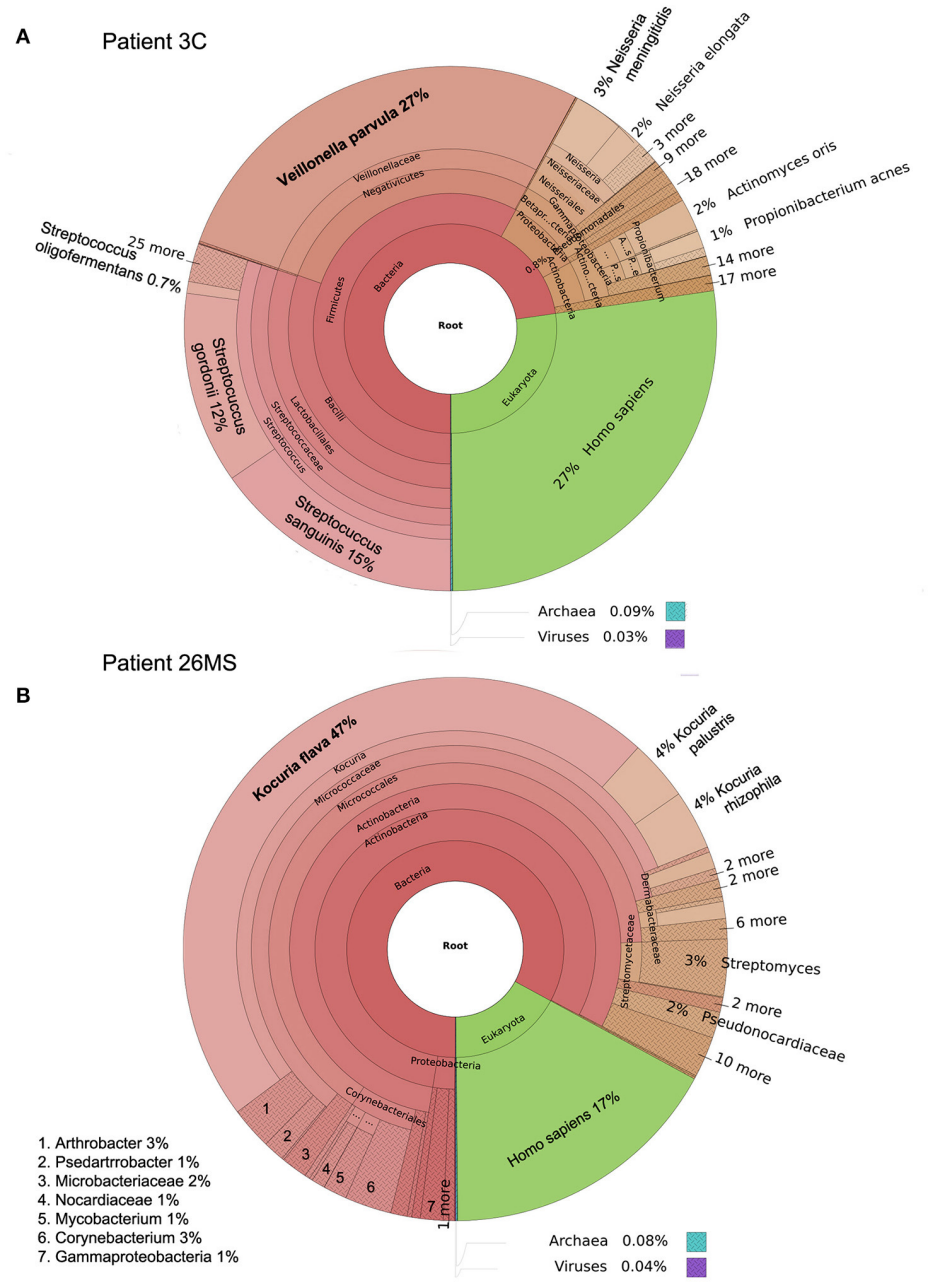
**Taxonomic classification of samples 3C (A)** and 26MS **(B)** performed by Kraken and plotted with Krona.

We initially attempted to extract DNA from 78 CSF samples from patients affected by MS or other neurological conditions. However, only 30 of those samples produced detectable levels of DNA, and we used them for the construction of metagenomics libraries (samples are described in Table 1). We also used non-measurable amounts of DNA from non-MS patients, and could obtain libraries suitable for sequencing from 15 samples, but it required a higher number of PCR amplification cycles. Hereafter, samples derived from multiple sclerosis patients are denoted by the letters “MS” while the rest are designated as “non-MS” or “control” samples. In a previous study to assess sensitivity of metagenomics for detection of microbial DNA in CSF, it was shown that libraries from cell cultures spiked in with only viral nucleic acids may yield up to ~4% of bacterial sequences, suggesting environmental contamination (Bukowska-Ośko et al., 2017). Using this frequency as a somewhat arbitrary threshold, we found that 4 out of 15 (~27%) non-MS samples contained more than 5% of bacterial reads (Figure 1, Figure S1, and Table 1), while only a single MS sample out of 28 (~3.6%) was found to harbor more than 5% of bacterial reads. Interestingly, all those samples correspond to female patients. The rest of libraries also had some bacterial reads, but at very low abundance, with 34 out of 43 libraries containing <1% bacterial reads (Tables 1, 2).

**Table 1:** **Description of samples and results of Kraken alignments in regards to the most abundant groups detected (human, bacteria, viruses, and archaea)**.

**Sample**	**Diagnostic**	**Gender**	**Human (%)**	**Bacteria (%)**	**Viruses (%)**	**Archaea (%)**
1C	6th nerve palsy	M	99.32	0.03	0.0005	0.0009
2C	Subarachnoid hemorrhage	M	98.88	0.20	0.004	0.006
**3C**	**Trigeminal neuralgia**	**F**	**27.46**	**73.48**	**0.03**	**0.09**
4C	Hypochondriasis	F	95.64	4.77	0.04	0.04
**5C**	**Chiari type I**	**F**	**83.37**	**15.93**	**0.009**	**0.05**
6C	Hypertension	M	99.7	0.20	0.002	0.001
**7C**	**FNS**	**F**	**91.99**	**6.77**	**0.02**	**0.09**
8C	Spasticity	M	98.57	1.49	0.01	0.002
9C	Idiopathic	M	99.36	0.31	0.01	0.006
10C	Parkinson	M	97.14	2.96	0.006	0.01
11C	Low back pain	M	99.96	0.11	0.003	0.005
**12C**	**Sciatica**	**F**	**81.25**	**18.26**	**0.09**	**0.07**
13C	IC hypertension	F	99.79	0.29	0.003	0.003
14C	IC hypertension	F	97.70	1.94	0.003	0.007
15C	IC hypertension	F	97.10	0.30	0.001	0.002
1MS	Multiple sclerosis	F	99.25	0.27	0.007	0.001
2MS	Multiple sclerosis	M	99.80	0.10	0.002	0.002
3MS	Multiple sclerosis	F	99.07	0.21	0.003	0.001
4MS	Multiple sclerosis	M	99.51	0.03	0.001	0.03
5MS	Multiple sclerosis	F	99.99	0.00	0.001	0.002
6MS	Multiple sclerosis	M	99.51	0.04	0.003	0.00
7MS	Multiple sclerosis	M	99.97	0.02	0.001	0.0002
8MS	Multiple sclerosis	F	99.13	0.09	0.0009	0.0005
9MS	Multiple sclerosis	M	99.51	0.10	0.002	0.002
10MS	Multiple sclerosis	M	99.96	0.31	0.002	0.001
11MS	Multiple sclerosis	M	99.81	0.02	0.00	0.01
12MS	Multiple sclerosis	F	99.67	0.05	0.0003	0.002
13MS	Multiple sclerosis	M	99.96	0.09	0.002	0.001
14MS	Multiple sclerosis	M	99.81	0.02	0.002	0.0005
15MS	Multiple sclerosis	F	99.73	0.03	0.0006	0.0008
16MS	Multiple sclerosis	F	99.81	0.11	0.001	0.00
17MS	Multiple sclerosis	F	99.45	0.07	0.002	0.001
18MS	Multiple sclerosis	F	99.78	0.11	0.002	0.002
19MS	Multiple sclerosis	M	99.96	0.01	0.0004	0.002
20MS	Multiple sclerosis	F	99.96	0.31	0.001	0.001
21MS	Multiple sclerosis	F	99.84	0.21	0.001	0.003
22MS	Multiple sclerosis	M	99.91	0.07	0.003	0.0002
23MS	Multiple sclerosis	M	99.96	0.11	0.002	0.001
24MS	Multiple sclerosis	M	99.98	0.04	0.0004	0.0008
25MS	Multiple sclerosis	F	99.99	0.03	0.001	0.0002
**26MS**	**Multiple sclerosis**	**F**	**16.99**	**83.25**	**0.04**	**0.08**
27MS	Multiple sclerosis	F	99.98	0.06	0.0003	0.0005
28MS	Multiple sclerosis	M	99.96	0.12	0.001	0.002

*Boldface rows correspond to samples that contained more than 5% of bacterial reads*.

**Table 2:** **Mean, standard deviation, and median abundance of human, bacterial, viral, and archaea DNA per group**.

**Group**	**Human**		**Bacteria**		**Viruses**		**Archaea**	
	**Mean [sd]**	**Median**	**Mean [sd]**	**Median**	**Mean [sd]**	**Median**	**Mean [sd]**	**Median**
Non-MS	91.1 [19.1]	97.7	8.5 [19.4]	1.5	0.02 [0.02]	0.006	0.03 [0.03]	0.006
MS	96.8 [15.6]	99.8	3.1 [15.7]	0.1	0.003 [0.007]	0.001	0.005 [0.02]	0.001

**Figure 2:**
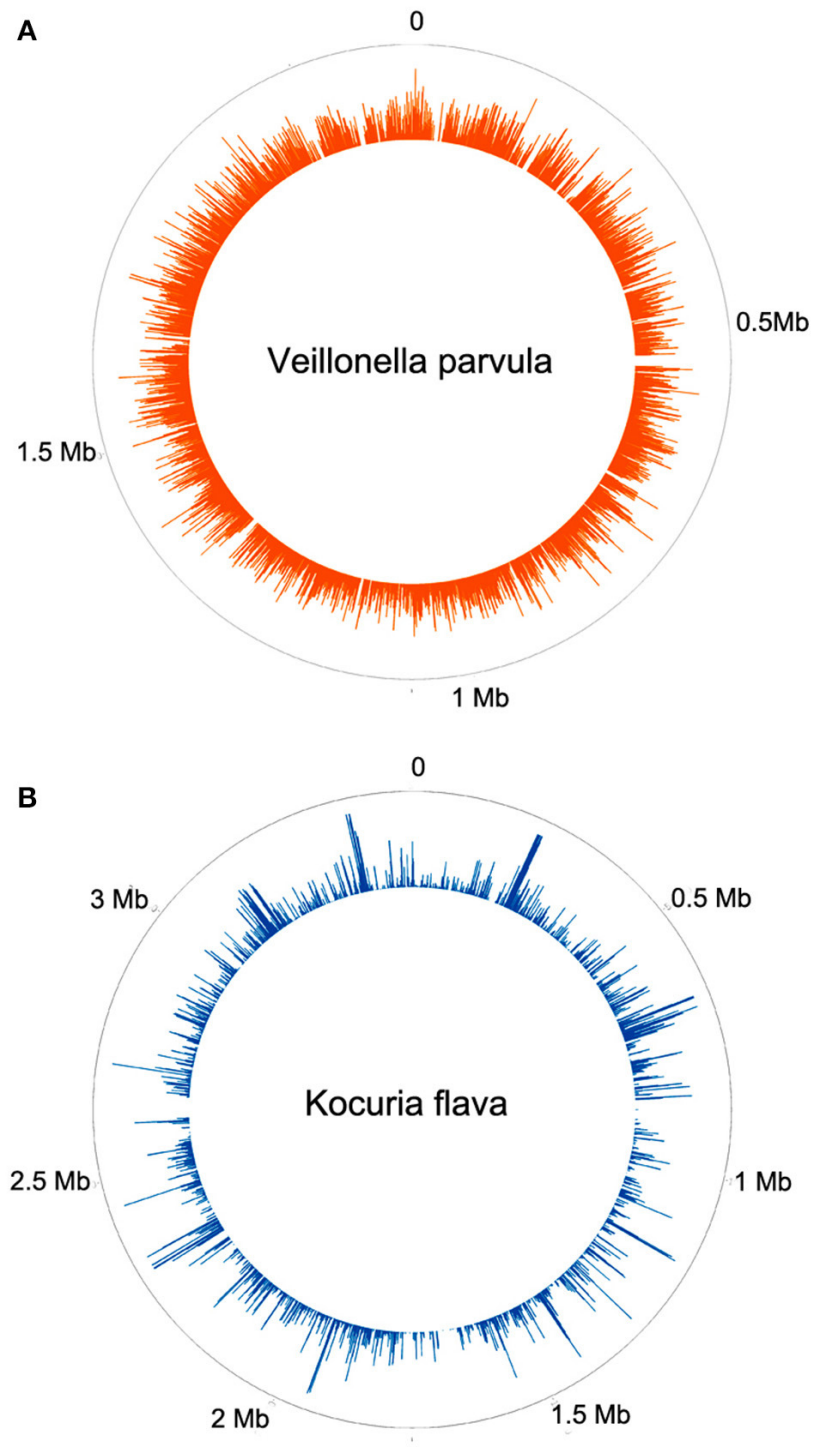
**Frequency histogram of relative abundance of reads aligning to different parts of the genome of ***Veillonella parvula*** (A)** or *Kocuria flava* **(B)**.

Two patients are of special interest (3C and 26MS). Patient 3C and 26MS harbored ~73 and 83% bacterial reads, respectively. The taxonomical bacterial profile of both samples was significantly different, excluding the possibility of cross-contamination between them (Figure 1). The sample from patient 3C was mainly populated with reads that resemble *Veillonella parvula*, and to a lesser extent *Streptococcus gordonii* and *S. sanguinis* and few other species of lower abundance (Figure 1A). Sample from patient 26MS was overwhelmingly populated by three species of *Kocuria* (*flava, palustris*, and *rhizophila*) and a series of non-abundant bacteria (Figure 1B). To exclude the possibility that an abundant and highly redundant sequence was producing spurious alignments to one or few loci in the genome of those two overrepresented bacteria, we plotted the relative abundance of reads along the whole genome of *V. parvula* (Figure 2A) and *Kocuria flava* (Figure 2B). The relatively homogeneous distribution of reads along the genome of those two bacteria suggests that those bacterial genomes were present in the sequenced pool of DNA, and excludes the possibility of artifactual alignments.

**Figure 3:**
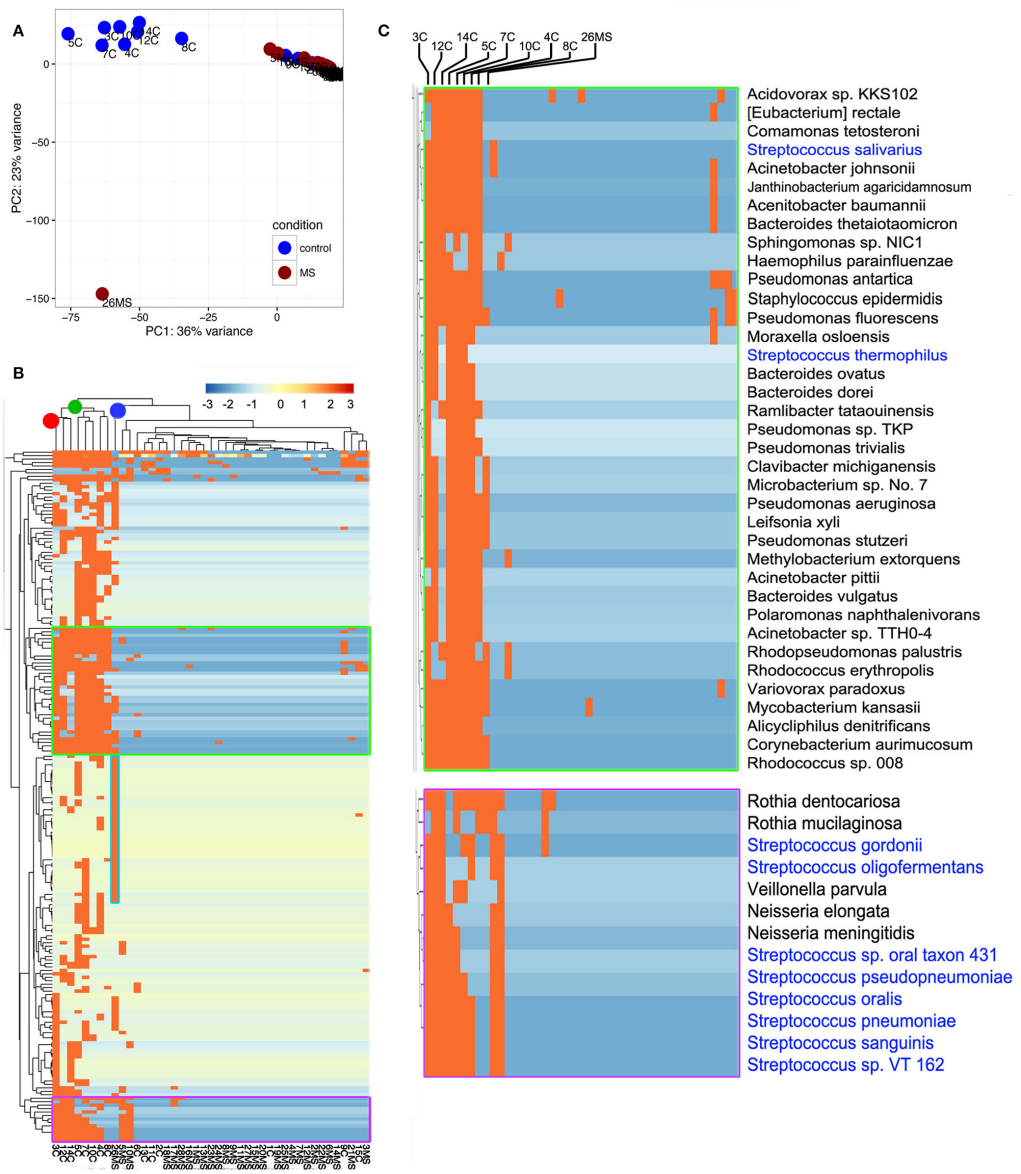
**Illustration of compositional relationship of samples as assessed by principal component analysis (A)** and hierarchical clustering **(B)**. In **(C)**, a close-up of some bacterial taxa frequently detected is shown.

To gain insights into the relationships between samples, we conducted principal component analysis (PCA; Jovel et al., 2016). PCA was conducted on any taxon that was detected in at least one sample (*n* = 559; Supplementary Table 1). The first Eigen vector of the PCA separated samples that contained more than 1% of bacterial reads from those that contained <1% (Figure 3A). This positioned samples 3C, 4C, 5C, 7C, 8C, 10C, 12C, and 14C on the left of the rest of control samples and somewhat proximal to each other (Figure 3A). The second Eigen vector of the PCA clearly separated sample 26MS from the rest of samples containing bacterial reads. Samples that contained <1% bacterial reads clustered together in the northeast quadrant of the plot, irrespectively of being MS or non-MS (Figure 3A). To refine our analysis, we conducted hierarchical clustering. In hierarchical clustering, an iterative algorithm positions samples with similar bacterial profiles closer on the branches of a dendrogram, while more dissimilar samples will branch apart (Jovel et al., 2016). The most ubiquitous bacterial species were *Propionibacterium acnes* and *Alteromonas mediterranea*, with the former bacterium previously found as contaminant in our libraries (Jovel et al., 2016). As deduced from the PCA analysis, all samples in the control group with more than 1% of bacterial reads clustered together in a single node (green node in upper dendrogram) with some minor subgroups (Figure 3B). For the samples in this cluster, the most-densely populated with bacterial reads (3C; red node on upper dendrogram; leftmost column in heatmap) might have acted as a contamination source, especially for samples that were sitting nearby in the same rack during library construction, since samples were ordered numerically on the rack. The fact that samples 4C, 5C, 7C, and 8C were also found to contain bacterial reads, supports the cross-contamination hypothesis. Accordingly, the profile of bacterial reads relative abundance depicted in the heatmap is similar, but not identical, between control samples than contained more than 1% of bacterial reads (Figure 3B). Noticeably, there are two conspicuous blocks of bacterial taxa that appear to be absent from most samples, but commonly represented in the control samples with more than 1% of bacterial reads. When such bacterial sets were zoomed in, a large number of bacterial species are evident, with the genera *Streptococcus, Pseudomonas*, and *Bacteroides* being among the most frequently detected (blue font in Figure 3C). The most abundant species in non-MS samples, *V. parvula*, appeared represented in five out of the eight control samples, but at high frequency only in sample 3C. As before, the MS sample harboring a large proportion of bacterial reads clearly separated from non-MS sample (blue node on upper dendrogram in Figure 3B). *K. flava*, the most abundant bacterial species in sample 26MS (73,796 reads) was found in some other samples (2MS, 18MS, 2C, 13C, 10MS, and 7C) but at a maximal abundance of 35 reads per library. Based on our previous experience, we regard that as cross-contamination. In addition to *K. flava*, sample 26MS contained a series of bacterial taxa of various abundances, which were only rarely seen in other samples (Figure 3B, cyan frame, Supplementary Table 1, colored red). In general, samples that are located close to each other on the wetlab rack can be considerably contaminated while trace cross-contamination may happen between all tubes, likely when DNA is aerosolized.

### Spurious viral hits

In addition to phage sequences, the most prevalent virus detected was the Human immunodeficiency virus 1 (HIV-1) and the herpes viruses 5 and 7. Although, at very low abundance (1–16 reads per library) the high frequency of HIV-1 sequences was rather suspicious and prompted us to inspect such hits more carefully. We extracted the raw reads that aligned to all viruses, and remove those ones corresponding to phages hits. The rest was aligned with BLAST against the whole “nt” database of NCBI. As expected, the HIV-1 hits were spurious since better hits either to lentiviral vectors or to a draft genome of the bacterium *Ralstonia solanacearum* were identified (Supplementary Table 2). On a closer inspection of the Ralstonia draft genome, we observed that the draft genome contains many sequences resembling Illumina adapters, which create artifactual alignments (Figure S2). A few sequences that were originally classified as cytomegalovirus (Human herpesvirus 5), Esptein-Barr virus (Human herpesvirus 4), parvoviruses or Paramecium chlorella bursaria virus were also confirmed as such during re-alignments (Supplementary Table 2, colored red). Thus, it is possible that such viruses are *bona fide* colonizers of CSF. In addition, sequences originally classified as Encephalomyocarditis virus were reclassified as Human simplex virus (Human herpesvirus 1). Some viral sequences that likely are associated with food, including Beet curly top virus and Iridoviruses also were confirmed during realignments.

### Bacterial and human cells were scarce in CSF

**Figure 4:**
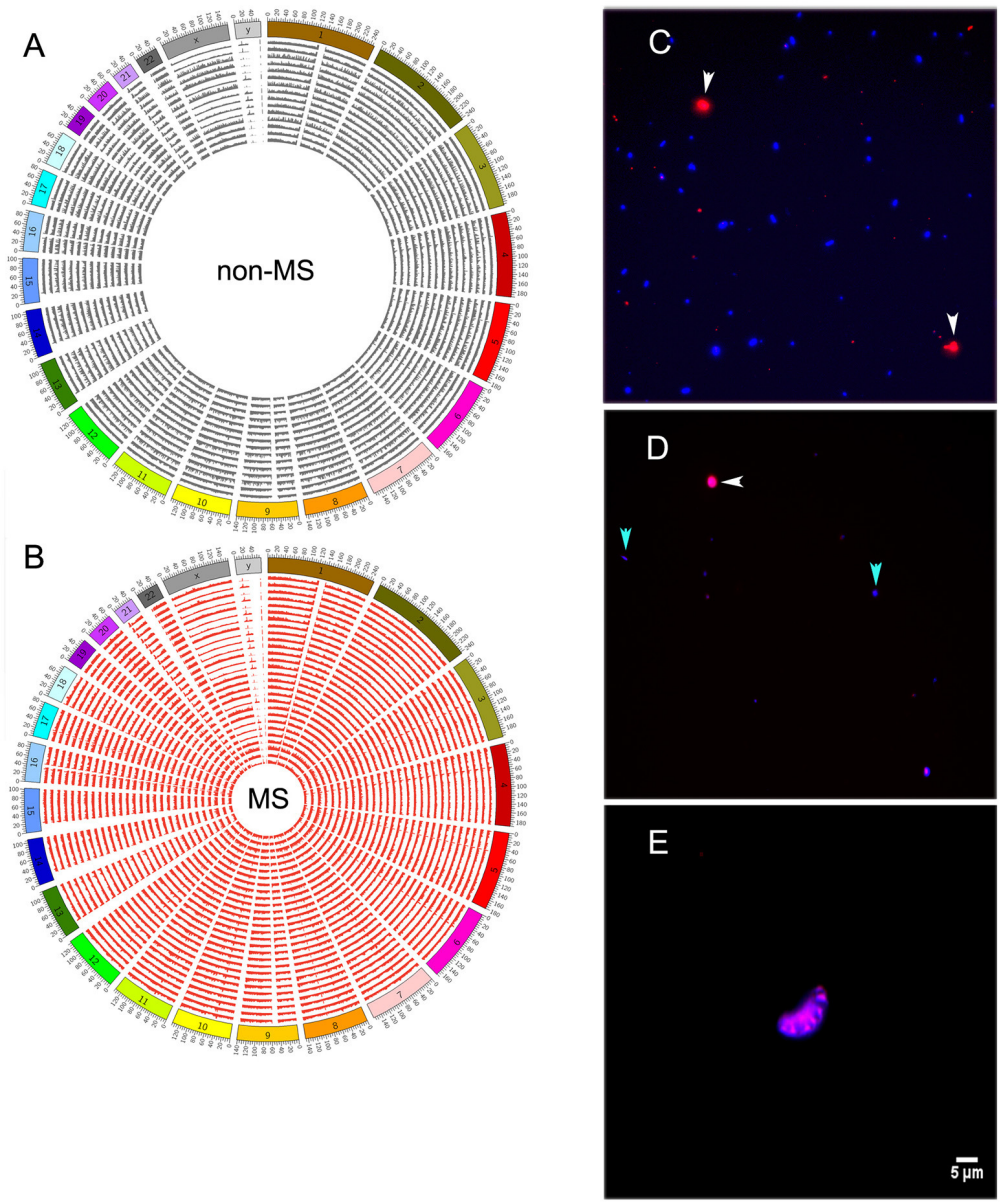
**Distribution of human reads along all chromosomes of the genome in non-MS (A)**, or MS **(B)** samples. **(C)** Staining of control bacterial (*Escherichia coli*) and human (THP-1 monocytes) cells with phalloidin and DAPI. Human cells are shown in red and bacterial cells are shown in blue. **(D)** A few bacterial cell-like structures and one human cell-like structure in sample 26MS. **(E)** A single cluster of putative human cells in sample 26MS from a different area of the slide. All other samples did not have any visible structure resembling human or bacterial cells. White and cyan arrowheads point to human and bacteria cells in **(C)** and to putative human and bacteria cells in **(D)**.

Reads mapping to the human genome spanned all chromosomes (Figures 4A,B). However, bacterial and human sequences detected in CSF need not be from intact circulating cells; they can also be from cell-free DNA. To distinguish between these two possibilities, we stained CSF with Alexa Fluor-594 phalloidin and DAPI. Phalloidin specifically stains human cells, while DAPI intercalates the DNA of both human and bacterial cells, which can ultimately be distinguished by size. We first prepared a control sample that was spiked with *Escherichia coli* and human THP-1 monocytes, and both human (red) and bacterial (blue) cells were readily detected after staining (Figure 4C). However, when we stained CSF samples, entities resembling stained bacteria or human cells were rarely found under fluorescence microscopy [Figure 4D (human and bacterial cells) and Figure 4E (human cells)]. Indeed, we screened all CSF samples described in Table 1, and with the exception of sample 26MS, we were unable to detect cell- or bacteria-like structures.

## Discussion

It has been a long-standing debate whether or not pathogens are involved in the etiology of multiple sclerosis (MS; Swanborg et al., 2003; Ramagopalan and Sadovnick, 2011; O'Gorman et al., 2012). Several lines of evidence suggest an infectious etiology. First, immunoglobulins with specificity against viruses or bacteria have been found in CSF from MS patients (Belbasis et al., 2015). Second, several bacteria, fungi, and viruses have been linked with demyelination (Johnson, 1994; Greenlee and Rose, 2000; Purzycki and Shain, 2010). Third, the differential geographical distribution of the disease (Giovannoni et al., 2006), the high incidence in developed countries (consistent with the hygiene hypothesis; Gilden, 2005), and changing incidence in subjects born in different seasons of the year (Willer et al., 2005), all might point toward pathogens as causal agents of MS.

A number of studies have reported the presence of pathogens in cerebrospinal fluid (CSF) from MS patients (Merelli et al., 1997; Moore and Wolfson, 2002; Swanborg et al., 2003; Stuve et al., 2004; Holmoy and Vartdal, 2005; Purzycki and Shain, 2010; Morandi et al., 2015), but the reproducibility of these experiments was often lacking (Casserly et al., 2007; Lindsey and Patel, 2008; Belbasis et al., 2015). Interestingly, a meta-analysis only found evidence of association of MS with smoking, IgG seropositivity to Epstein-Barr virus (EBV) nuclear antigen (EBNA) and infectious mononucleosis (Belbasis et al., 2015). Several experimental limitations might account for such a discrepancy. First, it may be that antibodies or PCR primers used for detection of pathogens lacked specificity and picked up background signal. Second, environmental contaminants are always a liability when conducting diagnostics in clinical samples. We applied metagenomics for the unbiased detection of microbial DNA in CSF samples from patients diagnosed with MS or other neurological conditions used as a comparison group.

Despite the limited size of the cohort of patients analyzed in this study (28 MS and 15 non-MS patients), our results suggest that CSF is often (but not always) free of bacteria and viruses, but always contain human DNA. However, we also need to entertain the hypothesis that some of the patients from our comparison group contained a higher frequency of microbial reads for some unknown reasons. The low background of bacterial reads observed across all libraries (<1%) likely represents cross-contamination events or environmental contamination that could have happened during sample collection, nucleic acids extraction, library preparation, or carry over in the sequencing instrument (i.e., from previous sequencing runs with libraries that contained the same barcodes used in this study). Contamination of libraries is a major concern when conducting next generation sequencing experiments (Jovel et al., 2016). Samples with more than 1% of bacterial reads likely reflect *bona fide* samples colonized with bacteria and/or bacterial DNA, with the highly abundant ones being less likely of representing contamination events.

Only one out of 28 MS samples contained a significant fraction of bacterial reads (83.25%), while eight out of 15 non-MS samples contained more than 1% of bacterial reads. On average, 19 and 24 PCR cycles were required for amplification of MS and non-MS libraries. The larger number of re-amplification cycles for non-MS samples means that such samples were manipulated more intensively than MS ones (i.e. each amplification PCR is followed by a clean up step with paramagnetic beads to remove low molecular weight material), which implies that non-abundant environmental contaminants may have been amplified and chances for cross-contamination increase.

In the non-MS sample with high load of bacterial DNA (3C), the most abundant species was *V. parvula*, a non-fermentative gram-negative coccus that is usually found colonizing the oral cavity, GI tract and vagina, but occasionally has been reported associated with meningitis (Bhatti and Frank, 2000) and other opportunistic infectious diseases in immunocompromised individuals (Strach et al., 2006). The most abundant species in sample 26MS was *K. flava*, an actinobacterium previously described as an airborne or a soil bacterium (Zhou et al., 2008; Achala et al., 2011). Thus, the association of such bacterium with MS is unclear. In summary, both *Veillonela parvula* in sample 3C and *Kocuria* spp. in sample 26MS are likely genuine bacteria colonizing the CSF of these patients. Literature reports on a putative role of bacteria on MS are rather discordant and hint to *M. pneumoniae, C. pneumonia*, and *S. aureus* as possible causal agents (Abramovitz et al., 1987; Brocke et al., 1993; Sriram et al., 1999; Libbey et al., 2014); this is incongruent with our main results.

A few viral sequences belonging to Epstein-Barr virus, cytomegalovirus and parvoviruses may be truly associated with CSF and may be involved in the etiology of MS, as previously suggested (Warren et al., 1977; Merelli et al., 1997; Moore and Wolfson, 2002; Swanborg et al., 2003; Stuve et al., 2004; Franciotta and Lolli, 2005; Gilden, 2005; Giovannoni et al., 2006). That only few viral-like sequences were detected in our libraries is not unexpected; this is dictated by the overwhelmingly much higher relative abundance of human and bacterial reads.

## Materials and methods

Study protocols and consent form was approved by the Health Research Ethics Board, University of Alberta (HREBA). All patients read and signed the consent form before sample collection.

### DNA extraction, construction of libraries, and sequencing

DNA from 48 multiple sclerosis (MS) patients and 30 patients with other neurological conditions was extracted from 200 μl of cerebrospinal fluid (CSF) using the Qiagen blood DNA mini kit, according to manufacturer's instructions. Only 28 MS samples produced DNA in amounts measurable by Qubit dsDNA HS assay kit (Invitrogen), all of them in the sub-nanogram per μl range. Only two non-MS samples produced DNA in measurable quantities (~0.3 ng/μl).

For construction of metagenomics libraries, the Nextera XT (Illumina) technology was used. In brief, 2 ng of DNA were tagmented (or whatever was available in 5 μl of DNA suspension) with 5 μl ATM for 5 min at 55°C. The tagmentation reaction was stopped with 5 μl of NT buffer and further incubation for 5 min at room temperature. Subsequently, the tagmentation products were PCR amplified in the presence of a distinct barcode for each sample, for 19 cycles in the case of MS, while non-MS samples needed on average 24 amplification cycles to obtain libraries in quantities sufficient for sequencing. After removal of primer dimers and low molecular weight materials, libraries were diluted to a concentration of 4 nM and pooled for sequencing. Sequencing was conducted in a MiSeq (Illumina) instrument, using a 600 cycles V3 kit and a workflow that includes adapter removal and demultiplexing.

### Bioinformatics analyses

Demultiplexed reads were aligned against a customized database using the ultrafast metagenomic classification package Kraken (Wood and Salzberg, 2014), and abundance estimates were generated with the package Bracken (Lu et al., 2016), which performs a Bayesian re-estimation of abundance after classification with Kraken. Plots were generated either with Krona (Ondov et al., 2011; hierarchical taxonomy plots) or using in-house R scripts. Reads that were reported by Kraken as virus hits were extracted from the row data and then aligned with BLAST against a recent version of the nt database of NCBI and top hits were retrieved for taxonomy comparison against Kraken classification by visual inspection of the corresponding alignments.

### Staining of bacterial and human cells

For each patient, 50 μl of CSF was added to 150 μl of RPMI media or *E. coli* HB101 (10^5^ CFU/ml) and THP-1 human monocytes (2.5 × 10^4^ cells) to 200 μl of RPMI on a 12 mm round coverslip (VWR) in a 24 well plate. The plate was spun at 1000 × g for 5 min and the supernatant removed. Coverslip was fixed with 4% paraformaldehyde (Fisher Scientific) for 15 min and washed thrice with 1X PBS. Coverslips were then blocked (2% Goat serum, 1% BSA) for 15 min. Actin was stained with Alexa Fluor-594 phalloidin (1:40 dilution, 0.1% Triton, 0.2% Goat Serum, and 0.1% BSA, Fisher Scientific); 4′, 6-diamidino-2-phenylindole (DAPI) (1:1000 dilution) was used for nuclear staining (0.1% Triton, 0.2% Goat Serum, and 0.1% BSA) and mounted using FluorSave (Calbiochem). Slides were analyzed using Zeiss Axio Observer.Z1 microscope with ZEN Imaging software (Carl Zeiss Canada Ltd., Toronto, ON, Canada) and the illustrations were edited using Image J.

## Author contributions

JJ, SO, and JP designed the experiments; MB conducted fluorescence microscopy; JJ, JP, and WW analyzed the data; KW contributed clinical samples; JJ, AM, and GW wrote the paper. Sequences of libraries described in this paper are publicly available at the SRA portal of NCBI under the accession number PRJNA354641.

## Funding

This work was supported by funding from Alberta Innovates Technology Futures-Innovates Centres of Research Excellence (AITF-iCORE) to GW.

### Conflict of interest statement

The authors declare that the research was conducted in the absence of any commercial or financial relationships that could be construed as a potential conflict of interest.

## Supplementary material

Figure S1 **Relative abundance of bacteria found in samples 12C (A)**, 7C **(B)**, and 5C **(C)**. For the sake of clarity, only the percentage of bacterial reads are presented. Human reads were overwhelmingly abundant in each sample (12C: 71.3%, 7C: 95.2%, 5C:83.4%). Others correspond to the pool of bacterial taxa, which individually constituted <1% of bacterial reads.

Figure S2 **Spurious virus alignments that also aligned to *Ralstonia solanacearum* are identical to Illumina adapters and transposase sequences. (A)** Sequences identical to the Illumina Nextera PCR primer i5 index N508. **(B)** Sequences identical to the Illumina Nextera PCR primer i7 index N711. **(C)** Sequences identical to the Illumina Nextera transposase sequence Read 2. **(D)** Sequences identical to the Illumina Nextera transposase sequence Read 1.

Supplementary Table 1 **Results of Kraken/Bracken classification and quantification per sample**. Some taxa with very low estimates of abundance (e.g., herpesviruses) are by default excluded by Bracken estimations.

Supplementary Table 2 **Results of realignment of eukaryotic viral hits from Kraken with BLAST against the “nt” database of NCBI**. In the “Query ID” column, the name of the sample, the name of the subject sequence originally identified by Kraken classification and the read name in the original FASTQ files are indicated.
