# Supplementary material for: Cerebrospinal Fluid in a Small Cohort of Patients with Multiple Sclerosis Was Generally Free of Microbial DNA
Source: Front Cell Infect Microbiol. 2017 Jan 6;6:198. doi: 10.3389/fcimb.2016.00198 (PMC5216046; doi:10.3389/fcimb.2016.00198)
Supplement: Figure S1 — Relative abundance of bacteria found in samples 12C (A), 7C (B), and 5C (C). For the sake of clarity, only the percentage of bacterial reads are presented. Human reads were overwhelmingly abundant in each sample (12C: 71.3%, 7C: 95.2%, 5C:83.4%). Others correspond to the pool of bacterial taxa, which individually constituted <1% of bacterial reads. [file DataSheet2.pdf]

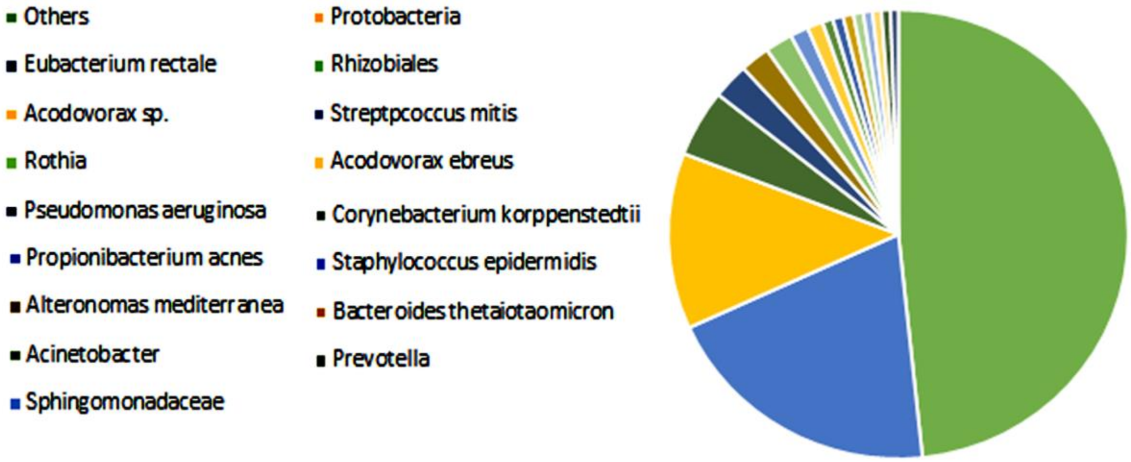

Others
Protobacteria
Eubacterium rectale
Rhizobiales
Acodovorax sp.
Streptpcoccus mitis
Rothia
Acodovorax ebreus
Pseudomonas aeruginosa
Corynebacterium korppenstedtii
Propionibacterium acnes
Staphylococcus epidermidis
Alteronomas mediterranea
Bacteroides thetaiotaomicron
Acinetobacter
Prevotella
Sphingomonadaceae

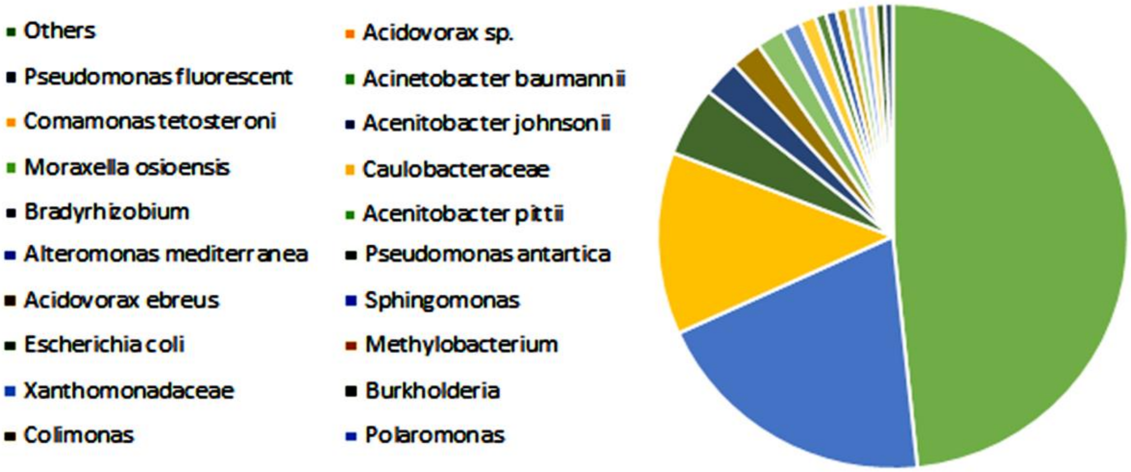

Others
Acidovorax sp.
Pseudomonas fluorescent
Acinetobacter baumannii
Comamonas tetosteroni
Acenitobacter johnsonii
Moraxella osloensis
Caulobacteraceae
Bradyrhizobium
Acenitobacter pittii
Alteromonas mediterranea
Pseudomonas antartica
Acidovorax ebreus
Sphingomonas
Escherichia coli
Methylobacterium
Xanthomonadaceae
Burkholderia
Colimonas
Polaromonas

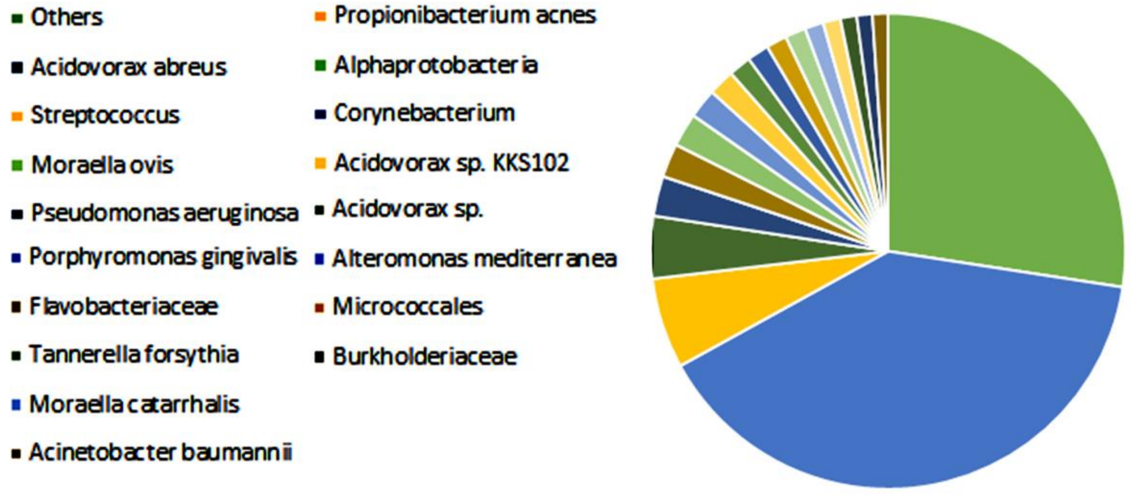

Others
Propionibacterium acnes
Acidovorax abreus
Alphaprotobacteria
Streptococcus
Corynebacterium
Moraella ovis
Acidovorax sp. KKS102
Pseudomonas aeruginosa
Acidovorax sp.
Porphyromonas gingivalis
Alteromonas mediterranea
Flavobacteriaceae
Micrococcales
Tannerella forsythia
Burkholderiaceae
Moraella catarrhalis
Acinetobacter baumannii

A

gnl|uv|NGB00734.1:1-51 Illumina Nextera PCR primer i5 index N508 (Oligonucleotide sequence copyright 2007-2012 Illumina,

Sequence ID: **Length:** 51 **Number of Matches:** 16

Range 1: 1 to 51 Graphics — ▼ Next Match ▲ Previous Match

Score	Expect	Identities	Gaps	Strand
102 bits(51)	2e-19	51/51(100%)	0/51(0%)	Plus/Minus

```
Query  3589187  GACGCTGCCGACGAAGGCTTAGGTGTAGATCTCGGTGGTCGCCGTATCATT  3589237
Sbjct  51       GACGCTGCCGACGAAGGCTTAGGTGTAGATCTCGGTGGTCGCCGTATCATT  1
```

Range 2: 1 to 51 Graphics — ▼ Next Match ▲ Previous Match ⇑ First Match

Score	Expect	Identities	Gaps	Strand
102 bits(51)	2e-19	51/51(100%)	0/51(0%)	Plus/Plus

```
Query  5212015  AATGATACGGCGACCACCGAGATCTACACCTAAGCCTTCGTCGGCAGCGTC  5212065
Sbjct  1        AATGATACGGCGACCACCGAGATCTACACCTAAGCCTTCGTCGGCAGCGTC  51
```

Range 3: 1 to 51 Graphics — ▼ Next Match ▲ Previous Match ⇑ First Match

Score	Expect	Identities	Gaps	Strand
102 bits(51)	2e-19	51/51(100%)	0/51(0%)	Plus/Minus

```
Query  5323965  GACGCTGCCGACGAAGGCTTAGGTGTAGATCTCGGTGGTCGCCGTATCATT  5324015
Sbjct  51       GACGCTGCCGACGAAGGCTTAGGTGTAGATCTCGGTGGTCGCCGTATCATT  1
```

B

gnl|uv|NGB00745.1:1-47 Illumina Nextera PCR primer i7 index N711 (Oligonucleotide sequence copyright 2007-2012 Illumina, Inc.

Sequence ID: **Length:** 47 **Number of Matches:** 110

Range 1: 1 to 47 Graphics — ▼ Next Match ▲ Previous Match

Score	Expect	Identities	Gaps	Strand
94.7 bits(47)	6e-17	47/47(100%)	0/47(0%)	Plus/Minus

```
Query  105833  CCGAGCCCACGAGACAAGAGGCAATCTCGTATGCCGTCTTCTGCTTG  105879
Sbjct  47      CCGAGCCCACGAGACAAGAGGCAATCTCGTATGCCGTCTTCTGCTTG  1
```

Range 2: 1 to 47 Graphics — ▼ Next Match ▲ Previous Match ⇑ First Match

Score	Expect	Identities	Gaps	Strand
94.7 bits(47)	6e-17	47/47(100%)	0/47(0%)	Plus/Minus

```
Query  908816  CCGAGCCCACGAGACAAGAGGCAATCTCGTATGCCGTCTTCTGCTTG  908862
Sbjct  47      CCGAGCCCACGAGACAAGAGGCAATCTCGTATGCCGTCTTCTGCTTG  1
```

Range 3: 1 to 47 Graphics — ▼ Next Match ▲ Previous Match ⇑ First Match

Score	Expect	Identities	Gaps	Strand
94.7 bits(47)	6e-17	47/47(100%)	0/47(0%)	Plus/Minus

```
Query  1739806  CCGAGCCCACGAGACAAGAGGCAATCTCGTATGCCGTCTTCTGCTTG  1739852
Sbjct  47       CCGAGCCCACGAGACAAGAGGCAATCTCGTATGCCGTCTTCTGCTTG  1
```

C

gnl|uv|NGB00726.1:1-34 Illumina Nextera transposase sequence Read 2 (Oligonucleotide sequence copyright 2007-2012

Sequence ID: **Length:** 34 **Number of Matches:** 119

Range 1: 1 to 34 Graphics — ▼ Next Match ▲ Previous Match

Score	Expect	Identities	Gaps	Strand
68.6 bits(34)	4e-09	34/34(100%)	0/34(0%)	Plus/Plus

```
Query  23  GTCTCGTGGGCTCGGAGATGTGTATAAGAGACAG  56
Sbjct  1   GTCTCGTGGGCTCGGAGATGTGTATAAGAGACAG  34
```

Range 2: 1 to 34 Graphics — ▼ Next Match ▲ Previous Match ⇑ First Match

Score	Expect	Identities	Gaps	Strand
68.6 bits(34)	4e-09	34/34(100%)	0/34(0%)	Plus/Plus

```
Query  110995  GTCTCGTGGGCTCGGAGATGTGTATAAGAGACAG  111028
Sbjct  1       GTCTCGTGGGCTCGGAGATGTGTATAAGAGACAG  34
```

Range 3: 1 to 34 Graphics — ▼ Next Match ▲ Previous Match ⇑ First Match

Score	Expect	Identities	Gaps	Strand
68.6 bits(34)	4e-09	34/34(100%)	0/34(0%)	Plus/Minus

```
Query  438642  CTGTCTCTTATACACATCTCCGAGCCCACGAGAC  438675
Sbjct  34      CTGTCTCTTATACACATCTCCGAGCCCACGAGAC  1
```

D

gnl|uv|NGB00725.1:1-33 Illumina Nextera transposase sequence Read 1 (Oligonucleotide sequence copyright 2007-2012

Sequence ID: **Length:** 33 **Number of Matches:** 28

Range 1: 1 to 33 Graphics — ▼ Next Match ▲ Previous Match

Score	Expect	Identities	Gaps	Strand
66.6 bits(33)	2e-08	33/33(100%)	0/33(0%)	Plus/Minus

```
Query  101365  CTGTCTCTTATACACATCTGACGCTGCCGACGA  101397
Sbjct  33      CTGTCTCTTATACACATCTGACGCTGCCGACGA  1
```

Range 2: 1 to 33 Graphics — ▼ Next Match ▲ Previous Match ⇑ First Match

Score	Expect	Identities	Gaps	Strand
66.6 bits(33)	2e-08	33/33(100%)	0/33(0%)	Plus/Minus

```
Query  3564517  CTGTCTCTTATACACATCTGACGCTGCCGACGA  3564549
Sbjct  33       CTGTCTCTTATACACATCTGACGCTGCCGACGA  1
```

Range 3: 1 to 33 Graphics — ▼ Next Match ▲ Previous Match ⇑ First Match

Score	Expect	Identities	Gaps	Strand
66.6 bits(33)	2e-08	33/33(100%)	0/33(0%)	Plus/Minus

```
Query  3589168  CTGTCTCTTATACACATCTGACGCTGCCGACGA  3589200
Sbjct  33       CTGTCTCTTATACACATCTGACGCTGCCGACGA  1
```
